# Intraocular lens power calculation in eyes with a shallow anterior chamber depth and normal axial length

**DOI:** 10.1371/journal.pone.0288554

**Published:** 2023-07-27

**Authors:** Yunjin Lee, Mee Kum Kim, Joo Youn Oh, Hyuk Jin Choi, Chang Ho Yoon

**Affiliations:** 1 Department of Ophthalmology, Seoul National University College of Medicine, Seoul, South Korea; 2 Department of Ophthalmology, Gachon University Gil Hospital, Incheon, Korea; 3 Department of Ophthalmology, Seoul National University Hospital, Seoul National University College of Medicine, Seoul, Korea; 4 Laboratory of Ocular Regenerative Medicine and Immunology (LORMI), Artificial Eye Center, Seoul National University Hospital Biomedical Research Institute, Seoul, South Korea; 5 Department of Ophthalmology, Seoul National University Hospital Healthcare System Gangnam Center, Seoul, South Korea; Alexandria University Faculty of Medicine, EGYPT

## Abstract

We compared the accuracy of three intraocular lens (IOL) calculation formulas in eyes with a shallow anterior chamber depth (ACD) and normal axial length (AXL) and control eyes. We retrospectively reviewed eyes with a shallow ACD (<2.5 mm from the corneal epithelium) with normal AXL (22.5≤AXL<24.0 mm) and controls (3.0≤ACD<3.5 mm and normal AXL). Prediction error (PE) and median absolute error (MedAE) were evaluated with SRK/T, Barrett Universal II (BUII), and Kane formulas after adjusting the mean PE to zero for all patients. Percentages of eyes achieving a PE within 0.25 to 1.00 D, and correlations between ACD, lens thickness (LT), and PE were analyzed. Thirty-five shallow ACD and 63 control eyes were included. PE in the shallow ACD group showed more hyperopic results with BUII and Kane but not with SRK/T compared to controls. Within the shallow ACD group, PE showed more hyperopic results in BUII and Kane compared to SRK/T. However, the standard deviation (SD) of PE among formulas was not different. In the shallow ACD group, SRK/T showed a higher percentage of PE within 0.25 D than BUII and Kane, but the percentages within 0.50 to 1.00 D were similar. PE was negatively correlated with ACD in BUII and Kane, and positively correlated with LT in all formulas. BUII and Kane may induce slight hyperopic shift in eyes with a shallow ACD and normal AXL. However, the performance of the three formulas was comparable in the shallow ACD group in terms of MedAE, the SD of PE, and the percentage of eyes achieving PE within 0.50 D.

## Introduction

In modern cataract surgery, the accurate intraocular lens (IOL) power calculation is a critical factor in achieving the desired target refractive outcome, which has become a key component as patients’ refractive expectations continue to rise [1, 2]. Advances in devices have significantly improved the accuracy of biometric measurements including the axial length (AXL), corneal refractive power (K), lens thickness (LT), and anterior chamber depth (ACD) in recent years [1–6]. Because the formula for calculating IOL power also significantly affects the prediction of refractive power, selection of the proper formula is essential to obtain the desired refractive target outcome.

Modern IOL calculation formulas show similar accurate prediction rates in eyes with a normal range of AXL, K, and ACD [1]. However, the accuracy of these formulas declines in eyes with short and long AXL, steep and flat K, or shallow and deep ACD. As demonstrated by previous studies, the prediction errors of IOL formulas showed myopic or hyperopic errors outside of the normal range [1].

Regarding the outcomes of cataract surgery in eyes with a shallow ACD, most studies have included patients with angle-closure glaucoma, short AXL, and pseudoexfoliation syndrome, as these factors are associated with shallow ACD [7–9]. However, angle closure glaucoma and pseudoexfoliation syndrome are likely to accompany zonulopathy that may induce additional errors in the effective lens position (ELP) estimated by IOL calculation formulas [10, 11].

Herein, we investigated the accuracy of IOL formulas in eyes with a shallow ACD and normal AXL (shallow ACD group) without comorbidities such as angle-closure glaucoma or pseudoexfoliation syndrome. First, we compared the accuracy of IOL calculation formulas (Barrett Universal II [BUII], Kane, and the SRK/T formulas) between patients with shallow ACD and normal control groups. Next, to evaluate the effect of ACD and LT on prediction error (PE), we computed the correlation analysis for each formula.

## Materials and methods

### Subjects and study design

A retrospective chart review was performed in patients who underwent uncomplicated cataract surgery at Seoul National University Hospital (SNUH) from November 2019 to December 2021. All procedures were conducted following the tenets of the Declaration of Helsinki, and the study design was approved by the Institutional Review Board of SNUH (IRB No. 2112-132-1284). Owing to the retrospective design of the study and the use of deidentified patient information, the review board waived the need for written informed consent.

The inclusion criteria were as follows: age of at least 20 years old, implantation of a Tecnis^®^ 1-piece monofocal IOL (ZCB00, Johnson and Johnson Vision, Jacksonville, FL, USA) with a 2.7 mm clear cornea incision, and a postoperative corrected distance visual acuity greater than 20/40. Based on previous studies [5, 6, 12], according to biometric measurement using an IOLMaster 700 (Carl Zeiss Meditec AG, Jena, Germany, software version 1.88), patients with an AXL between 22.5 mm and 24.0 mm and ACD (from the corneal epithelium) of 2.5 mm or less were classified as the shallow ACD group. Those with the same range of AXL and an ACD between 3.0 mm and 3.5 mm were classified as the normal control group.

The exclusion criteria were as follows: history of refractive surgery, keratoplasty, traumatic cataract, corneal diseases (keratoconus, bullous keratopathy, endotheliitis, opacity), angle closure glaucoma, zonular weakness, phacodonesis, retinal disease (choroidal neovascularization, retinoschisis, retinal detachment, macular holes, epiretinal membrane, exudative age-related macular degeneration), and additional limbal relaxing incision. If both eyes in one patient were eligible, the eye included was chosen randomly.

### Intraocular lens power calculation

Ocular biometry was performed in all eyes using an IOLMaster 700 which measures the optical path length from the corneal anterior surface to the retinal pigment epithelium as the AXL. Three IOL power calculation formulas were evaluated: SRK/T, BUII, and Kane. The SRK/T formula requires the A-constant of the IOL, power of the cornea, and AXL. The BUII formula requires input of the same factors as the SRK/T formula as well as the ACD, LT, and white-to-white corneal diameter as optional factors. The Kane formula needs the A-constant of the IOL, the power of the cornea, ACD, and sex as mandatory components and LT and center corneal thickness (CCT) as optional factors. The IOLMaster 700 software was used for calculations of the SRK/T formula using an A-constant of 119.3 provided by the User Group for Laser Interference Biometry and BUII formulas using a lens factor of 2.09 [13]. For the Kane formula, data were manually entered into the online calculator (https://www.iolformula.com/) by one investigator (YL) and the results were checked for plausibility by another investigator (CHY).

### Main outcome measures

Manifest refraction was measured postoperatively at 1–2 months. The main outcome measures were the PE calculated as the difference between the measured and predicted postoperative refractive spherical equivalent, the mean absolute error (MAE) calculated as the absolute mean deviation from the predicted postoperative refractive outcome, and the median absolute error (MedAE) calculated as the absolute median deviation from the predicted postoperative refractive outcome. To eliminate systemic error, we zeroed out the mean PE to zero. A negative value indicates a myopic prediction error that shows a more myopic result than the predicted refraction. In addition, the mean PE for each formula was zeroed out by adjusting the PE for each eye to eliminate the systematic error [14]. The percentages of eyes with PE within ± 0.25 diopters (D), ± 0.50 D, ± 0.75 D, and ± 1.00 D were calculated.

### Statistical analysis

Statistical analysis was performed using SPSS (version 22.0, IBM/SPSS Inc., Chicago, IL, USA), Prism (version 9.4.0, GraphPad Software, San Diego, CA, USA), and the R Project for Statistical Computing (https://www.r-project.org). Normal distribution of the data was analyzed with the D’Agostino–Pearson test. Demographics and biometry data between and within group(s) were compared with the independent *t*-test or Mann–Whitney U test. Comparisons among formulas were done with repeated measures (RM) one-way analysis of variance (ANOVA) with Tukey’s post hoc test or the Friedman test with Dunn’s post hoc test. The heteroscedastic method was used for comparisons of the standard deviation (SD) [14]. The Chi-square test or Fisher’s exact test were used to evaluate the differences between categorical variables between the two groups and the Cochran Q test among formulas was employed to evaluate the percentage of PE within ± 0.25 D, 0.50 D, 0.75 D, and 1.00 D. The Pearson’ correlation coefficient was used to examine the correlation of ACD and LT with PE. A p value lower than 0.05 indicated statistical significance.

## Results

Sixty-three (control group) and 35 (shallow ACD group) eyes of 98 patients were included in this study. Demographics and biometry data of the total group are listed in Table 1. In the control group, the mean AXL was 23.35 ± 0.32 mm, the mean ACD from the corneal epithelium and endothelium were 3.21 ± 0.14 mm and 2.67 ± 0.14 mm, respectively, and the mean LT was 4.40 ± 0.31 mm. In the shallow ACD group, the mean AXL was 23.25 ± 0.39 mm, the mean ACD from the corneal epithelium and endothelium were 2.30 ± 0.21 mm and 1.77 ± 0.21 mm, respectively, and the mean LT was 5.16 ± 0.30 mm. Compared to the control group, AXL and keratometry were comparable, the mean ACD was shallower (p < 0.001), and the mean LT was thicker (p < 0.001) in the shallow ACD group.

**Table 1:** Demographics and biometry information for the study population.

	Control group (n = 63)	Shallow ACD group (n = 35)	p value
Age ± SD (range), y	73.13 ± 5.52 (64–86)	74.49 ± 6.83 (63–89)	0.317^†^
Female (n, %)	38, 60.3%	26, 74.3%	0.164^‡^
Right eye (n, %)	32, 50.8%	16, 45.7%	0.630^‡^
AXL ± SD (range), mm	23.35 ± 0.32 (22.53–23.85)	23.25 ± 0.39 (22.51–23.99)	0.162^†^
Flat K ± SD (range), D	43.54 ± 1.04 (41.40–45.45)	43.19 ± 1.27 (40.15–45.49)	0.144^†^
Steep K ± SD (range), D	44.22 ± 1.09 (41.99–46.65)	44.00 ± 1.28 (41.96–46.86)	0.356^†^
Average K ± SD (range), D	43.89 ± 1.04 (41.80–46.03)	43.60 ± 1.25 (41.31–46.07)	0.226^†^
ACD from epithelium ± SD (range), mm	3.21 ± 0.14 (3.00–3.49)	2.30 ± 0.21 (1.63–2.49)	<0.001^†^
ACD from endothelium ± SD (range), mm	2.67 ± 0.14 (2.43–2.95)	1.77 ± 0.21 (1.12–1.98)	<0.001^†^
LT ± SD (range), mm	4.40 ± 0.31 (3.67–5.12)	5.16 ± 0.30 (4.59–6.00)	<0.001^†^
CCT ± SD (range), mm	538.79 ± 30.28 (480–622)	533.40 ± 31.05 (473–597)	0.405^†^

^†^Unpaired *t*-test

^‡^Chi-square test

ACD: anterior chamber depth; AXL: axial length; CCT: center corneal thickness; K: corneal power; LT: lens thickness; SD: standard deviation

**Fig 1:**
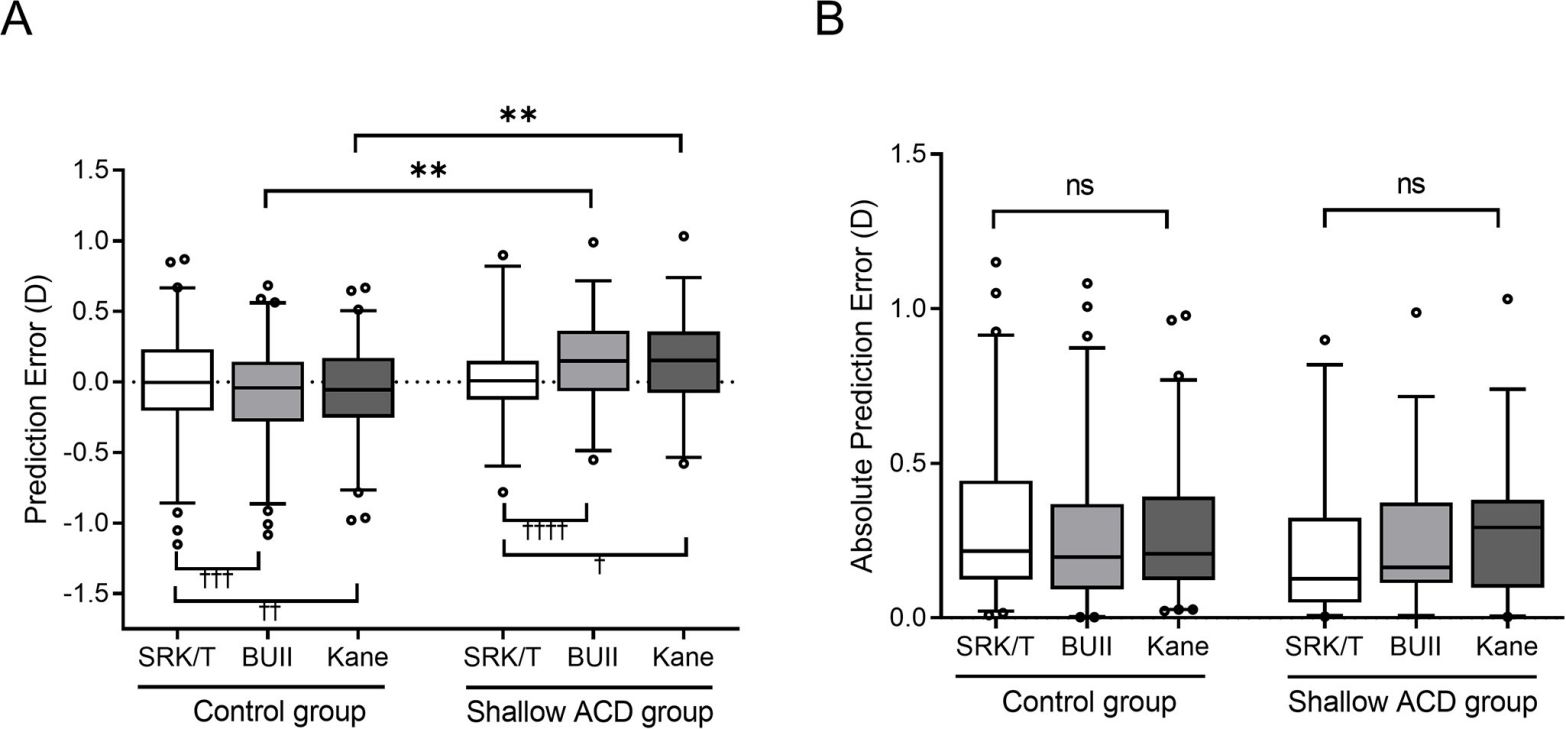
Box plot presentation of the (A) prediction error (PE) and (B) absolute PE of the three intraocular lens calculation formulas after adjusting the mean PE of all patients to zero. **p < 0.01, independent *t*-test, ^†^p < 0.05, ^††^p < 0.01, ^†††^p < 0.001, ^††††^p < 0.0001, repeated measures one-way analysis of variance with Tukey’s post hoc test, ns: not significant, The vertical lines on the whiskers represent the 5th and 95th percentiles. ACD: anterior chamber depth; BUII: Barrett Universal II.

The refractive outcomes of the three formulas between the two groups and within the groups are represented in Fig 1 and Table 2. After adjusting the mean PE to zero, the PE of the shallow ACD group compared to the control group showed a hyperopic shift in the BUII and Kane formulas (p = 0.002, p = 0.007, respectively; independent *t*-test) but not in the SRK/T formula (p = 0.982). However, there were no statistical differences in MedAE between the two groups. In the control group, PE showed more hyperopic results with the BUII formula than with SRK/T and Kane formulas (p < 0.05, one-way ANOVA with Tukey’s post hoc test, Fig 1A). In the shallow ACD group, PE showed more hyperopic results with the BUII and Kane formulas than with SRK/T formula (p < 0.05, one-way ANOVA with Tukey’s post hoc test; Fig 1A). However, there were no statistical differences in MedAE among the three formulas in both control and shallow ACD groups. (Fig 1B). For the heteroscedastic test, there was no statistically significant difference in the SD of PE among the three formulas in both the control and shallow ACD groups.

**Table 2:** The refractive outcomes of the three formulas after adjusting the mean prediction error (PE) to zero for all patients.

Group	Formula	Mean ± SD	MAE	MedAE
All	SRK/T	0.000 ± 0.377	0.273	0.196
	BUII	0.000 ± 0.358	0.266	0.190
	Kane	0.000 ± 0.358	0.279	0.211
Control	SRK/T	0.000 ± 0.407	0.304	0.216
	BUII	-0.079 ± 0.361	0.270	0.197
	Kane	-0.074 ± 0.351	0.276	0.207
Shallow ACD	SRK/T	0.000 ± 0.323	0.218	0.126
	BUII	0.142 ± 0.309	0.260	0.163
	Kane	0.133 ± 0.335	0.285	0.292

ACD: anterior chamber depth; BUII: Barrett Universal II; MAE: mean absolute error; MedAE: median absolute error; PE: prediction error; SD: standard deviation

**Fig 2:**
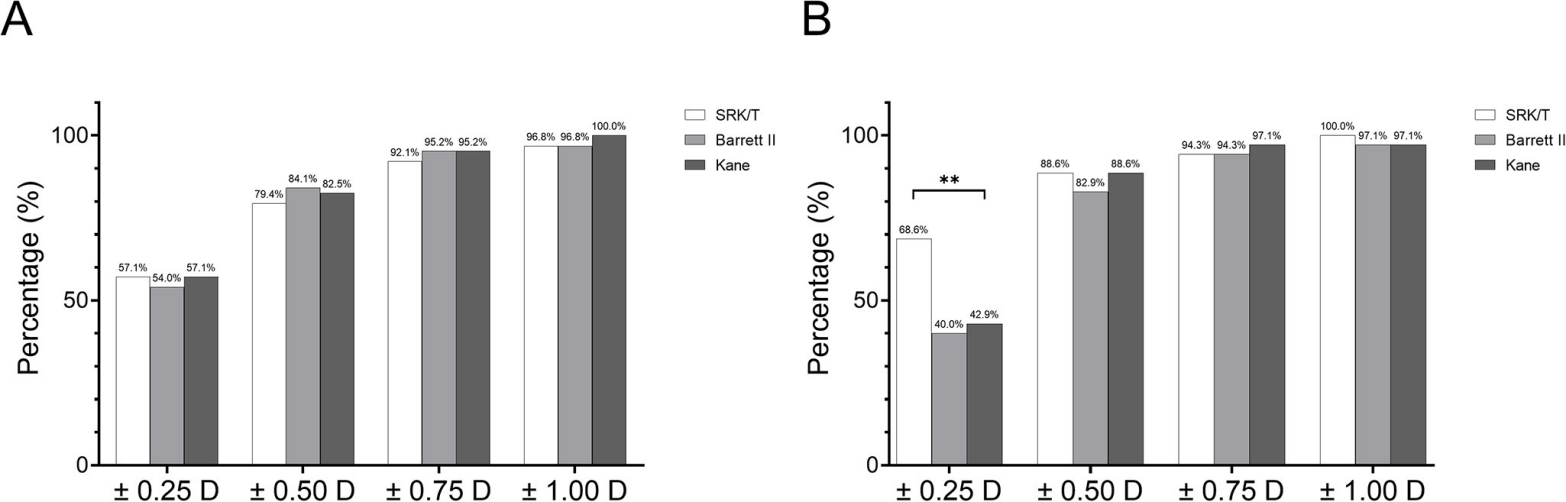
Percentage of eyes with prediction errors (PE) within ± 0.25 D, ± 0.50 D, ± 0.75 D, and ± 1.00 D in the control group (A) and shallow anterior chamber depth group (B) after adjusting the mean PE to zero. ** p < 0.01, Cochrane Q test, ACD: anterior chamber depth; D: diopter; PE: prediction errors.

The percentage of eyes within a certain range of PE is shown in Table 3 and Fig 2. The percentages of eyes within ± 0.25 D, ± 0.50 D, ± 0.75 D, and ± 1.00 D were not significantly different among the three formulas between the control and shallow ACD groups after adjusting the mean PE to zero (Table 3). Within the control group, no significant differences in PE were found among the three formulas in all ranges (all p > 0.05, Cochrane Q test, Fig 2A). However, in the shallow ACD group, SRK/T showed a superior PE within 0.25 D compared with the BUII and Kane formulas (p = 0.007, Cochran’s Q-test) (Fig 2B).

**Table 3:** Comparison of the percentage of cases within a given diopter range of prediction error (PE) between the two groups according to intraocular lens calculation formulas after adjusting the mean PE to zero.

PE	SRK/T			Barrett Universal II			Kane		
	Control group	Shallow ACD group	p value	Control group	Shallow ACD group	p value	Control group	Shallow ACD group	p value
± 0.25 D	57.1%	71.4%	0.162*	54.0%	57.1%	0.762*	57.1%	40.0%	0.104*
± 0.50 D	79.4%	91.4%	0.122*	84.1%	97.1%	0.091^†^	82.5%	97.1%	0.051^†^
± 0.75 D	92.1%	91.4%	>0.999^†^	95.2%	97.1%	>0.999^†^	95.2%	97.1%	>0.999^†^
± 1.00 D	96.8%	100.0%	0.536^†^	96.8%	100.0%	0.536^†^	100.0%	97.1%	0.357^†^

*Chi-square test

^†^Fisher’s exact test

ACD: anterior chamber depth; D: diopter; PE: prediction errors

**Fig 3:**
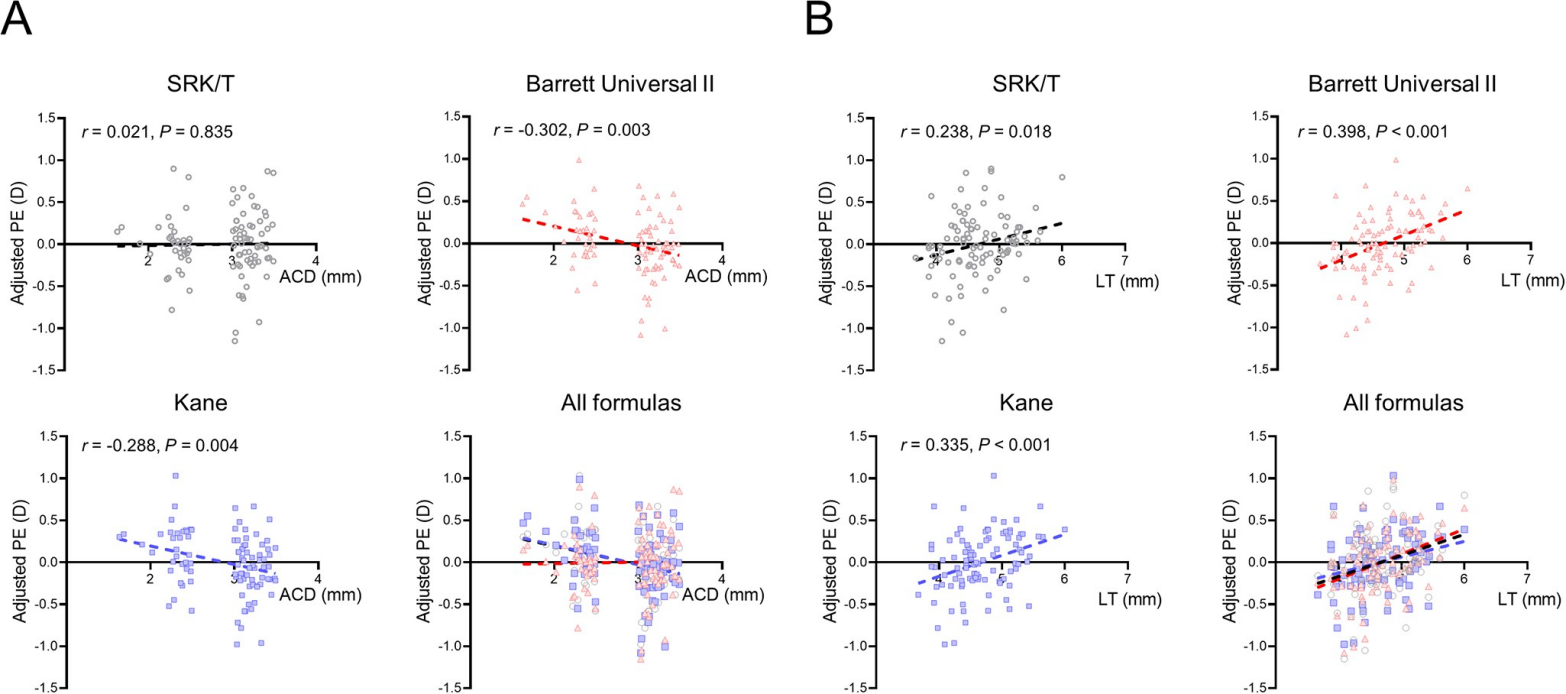
Correlation analysis of the association of preoperative anterior chamber depth (A) and lens thickness (B) after adjusting the mean prediction errors (PE) to zero. The ACD is negatively correlated with adjusted PE in the Barrett Universal II and Kane formulas, but no significant association is found in the SRK/T formula (Fig 3A). LT is positively correlated with adjusted PE in all three formulas (Fig 3B). ACD: anterior chamber depth; D: diopter; LT: lens thickness; PE: prediction error.

Finally, we performed correlation analysis between ACD and PE, and LT and PE (Fig 3). After adjusting the mean PE to zero, a negative correlation between preoperative ACD and PE was found in the BUII (r = -0.302, p = 0.003) and Kane formulas (r = -0.288, p = 0.004). However, there was no significant association between preoperative ACD and PE in the SRK/T formula (r = 0.021, p = 0.835) (Fig 3A). A positive correlation was found between LT and PE in all three formulas (all p > 0.05) (Fig 3B).

## Discussion

This study compared the three IOL formulas between the shallow ACD and control groups and within each group. Overall, all three formulas showed similar results in terms of MedAE, SD of the PE, and the percentage of PE within 0.5 to 1.0 D. However, the BUII and Kane formulas showed slight hyperopic errors in the shallow ACD group compared with the control group. In comparison within the shallow ACD group, the BUII and Kane formulas showed slight hyperopic error compared to the SRK/T formula.

The SRK/T formula is one of the third generation formulas and relies on only AXL and corneal keratometry for the estimation of postoperative ELP [15]. Retzlaff et al. developed the SRK/T formula in 1990, which was the most used 3rd generation formula in the past due to its excellent performance and high predictability [1, 9, 16, 17]. However, the BUII and Kane formulas are new generation formulas. The BUII was refined from the Barrett Universal I formula using a paraxial ray-tracing approach and uses AXL, K, ACD, LT, and white to white as variables [8, 18]. The Kane formula, introduced by Jack X Kane in 2018, utilizes several large data sets from selected high-volume surgeons [2]. This formula uses a combination of theoretical optics, thin-lens formulas, and ‘big data’ techniques, which enable its high predictions [2, 8, 18]. The Kane formula uses the AXL, K, ACD, LT, CCT values, and the gender of the patient to make its predictions [18]. The BUII and Kane formula became widely popular recently and are considered the most accurate formulas to predict postoperative refraction [1, 18, 19].

Shallow ACD was shown to be associated with age, female gender, hyperopia, small optic disk, short body stature, and chronic angle-closure glaucoma [20–22]. Primary angle closure and primary angle closure glaucoma eyes share a crowded anterior segment with a shallower ACD and a thicker and more anteriorly positioned lens [23]. In these patients, a shallow ACD may lead to inaccurate calculation of IOL power and highly variable refractive outcomes in cataract surgery [6, 20]. To date, there has been no definitive conclusion as to which formula best predicts outcomes in eyes with a shallow ACD and normal AXL. Although several studies have reported the effect of preoperative shallow ACD in the IOL formulation [5, 6, 9, 20, 24, 25], studies in these patients with a normal AXL are still limited. Thus, the purpose of this study was to evaluate the refractive outcomes of cataract surgery in eyes with a shallow ACD with normal AXL and to determine which of the commonly used IOL formulas (SRK/T, BUII and Kane) is the best for predicting postoperative refractive outcomes in these eyes.

In this study, we analyzed the accuracy of three widely used IOL calculation formulas because the BUII and Kane formulas include ACD and LT as variables and the SRK/T does not. To further clarify the results, we compared the shallow ACD group to eyes with a normal ACD and AXL as a control. AXL and keratometry were comparable between the shallow ACD and control groups, and adjusted PE of the SRK/T formula was not different between the two groups. However, the BUII and Kane formulas showed a hyperopic error of adjusted PE. Preoperative ACD was negatively correlated with adjusted PE in the BUII and Kane formulas, but not in the SRK/T formula. These results indicate that the BUII and Kane formulas, using ACD as a variable, predict that the shallower the ACD, the more forward the ELP is than its actual position. Yang et al. showed the effect of ACD on the refractive outcomes of the SRK/T, Holladay 1, Hoffer Q, and Haigis formulas [5]. The study reported that SRK/T showed the lowest refractive errors (RE) (0.02 ± 0.63 D) in the subgroup analysis of ACD < 2.5 mm (from the corneal epithelium) with an AXL range from 22.00 mm to 24.49 mm (n = 47), which is similar to this study. Hou et al. reported that the MedAEs (0.32 D to 0.37 D) for seven formulas (Kane, Hill-Radial Basis Function 3.0, Haigis, SRK/T, BUII, Hoffer Q, and Ladas super formula) were similar in eyes with primary angle closure disease with a normal AXL range from 22 mm to 25 mm [9]. The study reported that the number of hyperopic eyes would be the least when using the Hoffer Q formula followed by the SRK/T. Eom et al. reported that Haigis provided an equal MedAE of 0.40 D to Hoffer Q in eyes with a short AXL (≤ 22.0 mm) with a mean ACD of 2.63 mm (from the corneal epithelium) [24]. Miraftab et al. reported that the SRK-II formula induced less RE (-0.09 D) among the five formulas (Haigis, SRK-II, Hoffer Q, SRK/T, and Holladay 1) in eyes with an ACD ≤ 3.0 mm (from the corneal epithelium) and mean AXL of 22.91 mm [25]. A recently reported study by Yan el al. showed the BUII and Kane formulas showed good accuracy when comparing various IOL power formulas (BUII, Haigis, Hoffer Q, Hoffer QST, Holladay 1, Kane, and SRK/T) in eyes with an ACD < 3.0 mm (from the corneal epithelium) regardless of the AXL [6]. The study found that the accuracy of formulas such as the Haigis and Hoffer QST, which include ACD and not LT as variables, are affected by the lens vault (LV) measured by anterior optical coherence tomography. Wendelstein et al. reported that PEARL-DGS, Castrop, Okulix, and Kane formulas showed the lowest SD and medAE in eyes with a short AXL (20.98 mm, mean) with a mean ACD of 2.69 mm (from the corneal epithelium) when comparing 13 formulas including the SRK/T and BUII [26]. Result discrepancies between studies are likely due to differences in the age, underlying medical conditions, ethnicity, AXL, ACD, and keratometry of the enrolled patients, and the formulas analyzed.

Several studies have reported that LT or LV, defined as the vertical distance between the most anterior pole of the crystalline lens and the horizontal line connecting the two scleral spurs, is related with PE [1, 6, 21, 22, 27]. In this study, LT was positively correlated with PE in all three formulas. The result is similar to previous studies that have reported a tendency of hyperopic shift in eyes with higher LT across all formulas analyzed [1, 21]. In contrast, Yan et el. found that there are positive correlations between LV and PE predicted by five formulas (Haigis, Hoffer Q, Hoffer QST, Holladay 1, SRK/T) but not by Barrett and Kane formulas in eyes with a shallow ACD [6]. ACD and LT are closely related and the thicker the LT, the shallower the ACD. As these two factors influence PE, it seems that the hyperopic shift of PE was induced in the Barrett and Kane formulas.

The results of this study should be interpreted carefully because we only included eyes with a shallow ACD (< 2.5 mm) or normal ACD (3.0 mm ≤ ACD < 3.5 mm) with a normal AXL without any comorbidities such as angle closure glaucoma and pseudoexfoliation syndrome. In addition, we did not measure the preoperative LV. The importance of LV has been rising; therefore, further studies are needed to examine the relationship between LV and PE in various ocular conditions and formulas.

This study has some limitations. First, the sample size is small. Although a sufficient cohort group (6,501 patients, 11,700 eyes) was investigated, only 35 eyes satisfied the criteria of the shallow ACD and normal AXL group with no comorbidities. Second, not all available IOL calculation formulas were compared in this study. Comparing more formulas would be helpful, though this also creates multiple comparison issues and makes it difficult to address statistical significance. Therefore, we utilized the formulas commonly used in eyes with a normal AXL. Further large-scale studies with more IOL calculation formulas are needed. Third, this study included only a single type of IOL (Tecnis^®^ ZCB00). Caution should be exercised when applying the results of this study to other types of IOLs. Fourth, the IOLMaster 700 measures AXL using a single refractive index. Therefore, AXL measurement errors are more likely to occur in eyes with a shallow ACD because the crystalline lens with a high refractive index is thick. Therefore, AXL calculation with different refractive indexes may reduce error in the shallow ACD group.

In conclusion, The BUII and Kane formulas showed slight hyperopic errors in the shallow ACD group. However, SRK/T, BUII, and Kane formulas provided comparable refractive outcomes in terms of MedAE, SD, and the percentage of PE within 0.5 D considering that 0.50 D is the currently used IOL power step.
